# Exercise-Induced Cardiac Fatigue in Recreational Ultramarathon Runners at Moderate Altitude: Insights From Myocardial Deformation Analysis

**DOI:** 10.3389/fcvm.2021.744393

**Published:** 2022-01-24

**Authors:** Sebastián Wolff, José M. Picco, Leonel Díaz-González, Pedro L. Valenzuela, Emanuel Gonzalez-Dávila, Alejandro Santos-Lozano, Pablo Matile, David Wolff, Araceli Boraita, Alejandro Lucia

**Affiliations:** ^1^Wolff Cardiology and Sport Medicine Institute, Mendoza, Argentina; ^2^Cardiology Department, CEMTRO Clinic, Madrid, Spain; ^3^Cardiology Department, La Paz Hospital, Madrid, Spain; ^4^Faculty of Sport Sciences, Universidad Europea de Madrid, Madrid, Spain; ^5^I+HeALTH, European University Miguel de Cervantes, Valladolid, Spain; ^6^Physical Activity and Health Research Group (“PaHerg”), Research Institute of the Hospital 12 de Octubre (“Imas12”), Madrid, Spain; ^7^Matile Laboratory, Mendoza, Argentina; ^8^Department of Cardiology, Sports Medicine Center, Spanish Sports Health Protection Agency, Consejo Superior de Deportes, Madrid, Spain

**Keywords:** ventricular function, myocardial strain, myocardial deformation, athlete's heart, heart fatigue, ultra-marathon

## Abstract

**Background:**

Controversy exists on the actual occurrence of exercise-induced cardiac fatigue (EICF) with ultraendurance exercise, as well as on whether factors such as age or training status might predispose to this condition. The present study aimed to assess the occurrence of EICF among recreational ultramarathon runners, as well as to determine potential predictive factors.

**Methods:**

Nineteen male recreational runners (42 ± 12yrs) participated in a 55-km trial race at moderate altitude (1,800–2,500 m). Participants were evaluated before and after the race using Doppler echocardiography and myocardial deformation analysis. EICF was determined as a reduction >5% of either left ventricular global longitudinal strain (LVGLS) or right ventricular free wall strain (RVFWS). Demographical (age, body mass index), training (training experience, volume and intensity), competition (finishing time, relative intensity) and biochemical variables (blood lactate, creatine kinase [CK] and CK-MB) were assessed as predictors of EICF.

**Results:**

A significant reduction in LVGLS (20.1 ± 2.1% at baseline vs. 18.8 ± 2.4% at post-race, *p* = 0.026), but not in RVFWS (27.4 ± 7.0 vs. 24.6 ± 5.3%, *p* = 0.187), was observed after the race. EICF was present in 47 and 71% of the participants attending to the decrease in LVGLS and RVFWS, respectively. No associations were found between any of the analyzed variables and EICF except for age, which was associated with the magnitude of decrement of RVFWS (*r* = 0.58, *p* = 0.030).

**Conclusions:**

Ultramarathon running at moderate altitude seems to induce EICF in a considerable proportion of recreational athletes.

## Introduction

Although some controversy exists (1), strenuous endurance exercise might produce not only skeletal but also cardiac muscle fatigue, a condition known as “exercise-induced cardiac fatigue” (EICF) that is reflected by a transient decline in left (LV) or right ventricular (RV) function after exertion in otherwise healthy people (2–4). Although EICF can be assessed with conventional echocardiographic parameters such as LV ejection fraction (LVEF) (5), myocardial deformation imaging (i.e., global longitudinal strain [GLS] obtained through speckle tracking echocardiography) is emerging as a more reliable and sensitive measure (6, 7).

Different types of exercise might induce EICF, especially ultraendurance races (8–10). In addition, although controversy exists (11), recent evidence suggests that ultraendurance running at high altitudes can increase the risk of exercise-induced electrocardiographic alterations suggestive of EICF (i.e., longer QT duration and premature ventricular beats) (12). This is of relevance given the growing popularity of these events, which engage not only well-trained athletes but also recreational participants and are sometimes held in difficult environmental conditions (13). However, controversy exists on the actual occurrence of EICF in recreational runners after ultraendurance exercise (14), and the evidence on races conducted at high altitudes is particularly scarce. On the other hand, different factors such as age, training status and performance level have been associated with the risk of electrocardiographic alterations observed after ultraendurance exercise in competitive athletes (15), but it remains to be determined whether these factors might predispose to the occurrence of EICF in recreational runners.

In the present study we aimed to assess the occurrence of EICF through myocardial deformation analysis among healthy recreational runners who participated in an ultraendurance race held under difficult environmental conditions (i.e., moderate-high altitude). We also studied potential predictive factors.

## Methods

### Participants

Nineteen recreational male athletes (age: 42 ± 12 years; body mass index: 24.9 ± 3.0 kg·m^−2^; mean office [“clinic”] systolic and diastolic blood pressure upon recruitment: 117 ± 6 mmHg and 75 ± 8 mmHg, respectively [all ≤ 130/80 mmHg]; 9 ± 7 years of training experience in trail running) volunteered to participate in the study. Inclusion criteria were not presenting any cardiopulmonary disease, being a recreational (i.e., not professional) runner, and being able to finish an ultra-endurance trail race. Participants provided written informed consent after having the procedures explained. The study was conducted in accordance with the Declaration of Helsinki and its later amendments, and was approved by the local Ethics Committee.

### Study Design

Doppler echocardiography and myocardial deformation (“strain”) analysis was performed 2 days before the race and after 48–72 h of active rest or gentle training (i.e., “baseline") and immediately after (post-race) the 2019 *Cruce Mendoza* race, a self-supply 55-km trail race (total elevation gain of 2,500 m) where runners are largely responsible for their own hydration (i.e., with only three hydration posts held by the race organizers, at 10, 25, and 35 km, respectively). The race began in Villavicencio at a height of 467 meters above sea level (m a.s.l.) at an average temperature of 17°C and humidity of 12%. The race reached its highest point at the *Cruz de Paramillos* at 3,000 m a.s.l. with a temperature of −8°C, and finished near the Uspallata valley (2,039 m a.s.l.) where temperatures was around 7°C.

Participants were required to register all their training sessions during the 6 months previous to the race by means of their sport watches, and blood analyses were performed after the race.

### Echocardiography Measures

Two experienced cardiologists performed all procedures (JP & EG, with only one of them in charge of both baseline and post-race assessments in a given participant, and with each cardiologist assessing ~50% of the participants) using a Vivid-I ultrasound device (General Electric Vingmed, Milwaukee, WI). Images were analyzed off-line using post-processing software (Echo-Pac, GE Medical v201; Horten, Norway). The left (LV) and right ventricle (RV) diameter were measured in 2D on the left parasternal long axis and in the apical four-chamber plane (4C) at the tricuspid annulus, respectively. Pulsed wave Doppler imaging was used to determine ventricular filling pressures. Mitral inflow velocity was assessed by pulsed wave Doppler from the 4C view, positioning the sample volume at the tip of the mitral leaflets. Deceleration time of the E wave was measured as the interval from the peak E wave to its extrapolation to the baseline. E/e′ ratio was calculated as E wave divided by e′ velocities. The isovolumic relaxation time of the RV [IRTRV, reference values close to 0 milliseconds (16)] was measured using tissue Doppler imaging, immediately above the tricuspid ring on the free wall of the RV. The size of the left and right atrium was determined through the indexed atrial volume, performed in 4C and apical 2C and by area in the apical 4C, respectively. The systolic excursion of the tricuspid annulus (TAPSE) was recorded using M-mode, measuring from the annular base to the maximum excursion. To estimate preload and afterload parameters, end-diastole and end-systole volumes indexed by body surface area were determined. Ventricular volumes, LVEF, cardiac output (CO) and GLS of the LV (LVGLS) were determined by automatic detection of the endomyocardial border with the least possible intervention by the operator (“automatic functional imaging” or “AFI”). The parameters of ventricular torsion, as well as the RV free wall strain (RVFWS) were obtained using the Q-analysis post-processing software (GE Medical). Left atrial strain was measured by Q-analysis using the arithmetic mean strain on the apical 4C and 2C. Considering the results of recent research showing that, regardless of echocardiographic training, the inter-observer variability is much lower for LVGLS assessment compared to LVEF and below 5% (i.e., mean relative difference between expert and untrained observer of 3.61 and 6.56%, respectively) (6), we defined EICF as relative decrease from baseline to post-race of LVGLS or RVFWS values above 5%.

### Potential EICF Predictors

Participants' training data during the 6 months preceding the race were collected using the information registered by their sport watches (Garmin^®^, Tom-tom^®^, Samsung^®^). The information collected included different markers of training volume (training hours per week, km per month, and total elevation gain per session) and intensity (average heart rate [HR], expressed as a percentage of the theoretical maximum heart rate [HRmax, determined as 220 minus age]). We also recorded the mean HR during the race and the time needed for its completion. Body mass was assessed before and after the race, and the change (as a %) was analyzed as a marker of dehydration. Finally, serum samples were obtained from the antecubital vein immediately after the race for the analysis of blood lactate, creatine kinase (CK) and CK-myocardial band (CK-MB) (Architect c4000 clinical chemistry analyzer, Abbot laboratories, Chicago, IL). The upper reference values for CK and CK-MB were 110 and 25 U/L, respectively.

### Statistical Analysis

Data are shown as mean ± standard deviation (SD). The Wilcoxon signed-rank test was used to analyze changes from baseline to post-exertion. Univariate logistic regression analyses were used to assess the association between potentially predictive variables and the occurrence of EICF. Linear regression analyses were also performed to assess the association between these variables and the magnitude of impairment of cardiac function (i.e., relative change from baseline to post-race in LVGLS or RVFWS) as a continuous variable. In addition, the Fisher's exact test was used to compare the proportion of EICF occurrence depending on age (i.e., participants aged ≥ or <45 years, respectively). Analyses were performed using SPSS v23 statistical package (IBM statistics; Chicago, IL) with the threshold for statistical significance set at <0.05.

## Results

During the 6 months preceding the race participants trained on average 8.6 ± 3.6 h per week, covering 173 ± 52 km per month and 1,236 ± 598 m of total elevation gain per session at an intensity of 136 ± 11 bpm (i.e., 78 ± 5% of HRmax). Participants completed the 55-km trail race in 426 ± 62 min (i.e., 27% slower performance time than the winner), at a mean HR of 138 ± 8 bpm (79 ± 4% of HRmax), and lost 1.8 ± 2.7% of their body mass during the race. CK and CK-MB levels at the end of the race averaged 652 ± 345 U/L and 52 ± 22 U/L, respectively, whereas blood lactate levels averaged 4.4 ± 1.4 mmol/L.

**Figure 1:**
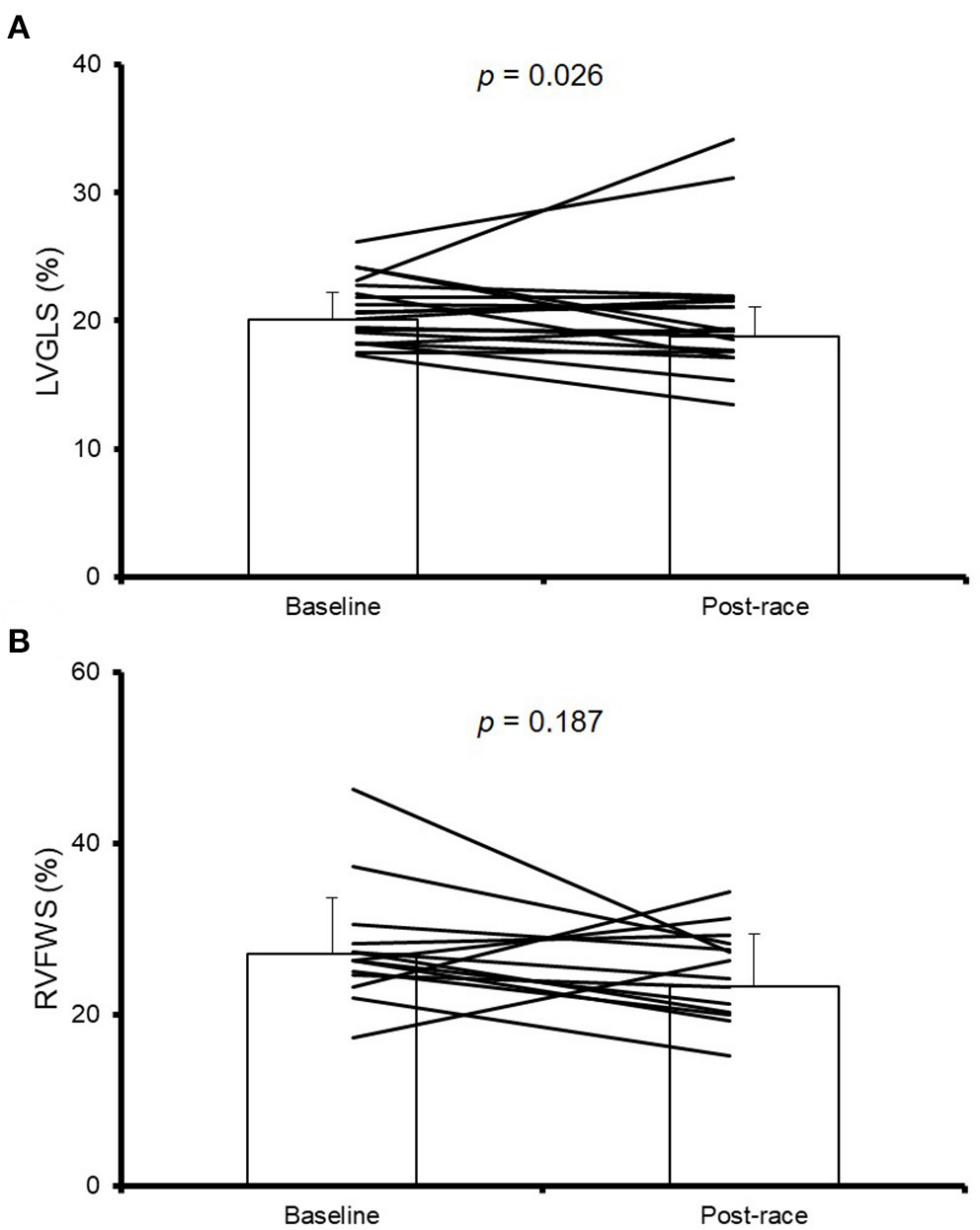
Myocardial deformation results. Individual and mean data (± standard deviation) results of left ventricular global longitudinal strain (LVGLS) **(A)** and right ventricular free wall stress (RVFWS) **(B)** at baseline and post-race. RVFWS could be assessed in 14 of the 19 participants.

Post-race echocardiographic evaluations were conducted 19 ± 10 mins after the participants crossed the finish line. The occurrence of EICF based on complete baseline and post-race measures of LVGLS and EVFWS, respectively, could be assessed in 19 (100% of total) and 14 (74%) participants. The proportion of participants with complete data showing EICF (i.e., relative decrease in post-race values compared to baseline >5%) was of 47 and 71% for LVGLS and RVFWS measures, respectively. The proportion of participants with a very high relative decrease (i.e., >15%) in LVGLS and RVFWS was of 32 and 54%, respectively. In turn, a significant reduction was found for mean values of LVGLS after the race (−6.5% on average, *p* = 0.026) but not for those of RVFWS (*p* = 0.187), with considerable inter-individual variability, especially for RVFWS (Figure 1).

The mean values of the following echocardiographic variables decreased significantly after the race (Table 1): LV-end-diastolic volume (LVEDD) and indexed LVEDD, indexed left atrial volume, RV end-diastolic diameter, LEVF and TAPSE. By contrast, a significant increase was found for E/A, e' and E/e'.

**Table 1:** Baseline and post-race echocardiographic results.

	**Baseline**	**Post-race**	***P-value* (Wilcoxon test)**
**Cardiac chamber dimensions**			
LVEDD (mm)	49.7 ± 3.3	47.1 ± 3.2	**0.009**
iLA volume (ml/m^2^)	33.9 ± 6.6	28.3 ± 4.5	**0.004**
iLVEDV (ml/m^2^)	57.9 ± 7.9	52.0 ± 8.2	**0.010**
iLVESV (ml/m^2^)	23.6 ± 4.2	22.8 ± 4.2	0.647
RVEDD (mm)	38.0 ± 5.0	41.3 ± 6.7	**0.020**
RA area (cm^2^)	18.2 ± 3.9	18.9 ± 3.1	0.646
**Ventricular systolic function**			
CO (L/min)	3.9 ± 0.9	4.2 ± 0.7	0.144
LV S' (cm/s)	10.6 ± 3.1	11.3 ± 3.1	0.622
LVEF (%)	60.7 ± 4.9	57.6 ± 3.0	**0.007**
Twist (°)	9.0 ± 4.2	9.7 ± 3.7	0.638
TAPSE (mm)	27.6 ± 2.0	24.6 ± 4.2	**0.002**
RV S' (cm/s)	10.6 ± 3.1	11.3 ± 3.1	0.443
**Ventricular diastolic function**			
IRTRV (ms)^*^	-	100 ± 36	
E/A	1.11 ± 0.32	1.37 ± 0.50	**0.025**
e' (cm/s)	12.3 ± 2.9	14.1 ± 3.4	**0.032**
E/e'	6.2 ± 2.1	4.5 ± 1.2	**0.001**
Left atrial strain (%)	29.4 ± 4.6	28.0 ± 6.2	0.234

*Data are presented as mean ± SD. Significant p-values are in bold*.

* *Reference values of 0 ± 3 were taken as baseline measures for IRTRV. CO, cardiac output; e', tissue Doppler e' wave at the mitral annulus; iLA, indexed left atrium volume; iLVEDV, indexed left ventricular end-diastolic volume; iLVESV, indexed left ventricular end-diastolic volume; IRTRV, isovolumic relaxation time of the right ventricle; LVEDD, left ventricular end-diastolic diameter; LVEF, left ventricular ejection fraction; LVGLS, left ventricular global longitudinal strain; LV S', LV tissue Doppler S' wave at the left mitral annulus; RA, right atrium; RVEDD, right ventricular end-diastolic diameter; RVFWS, right ventricular free wall strain; RV S' = RV tissue Doppler S' wave at the tricuspid annulus; TAPSE, tricuspid annular plane systolic excursion; twist, left ventricular systolic torsion*.

**Figure 2:**
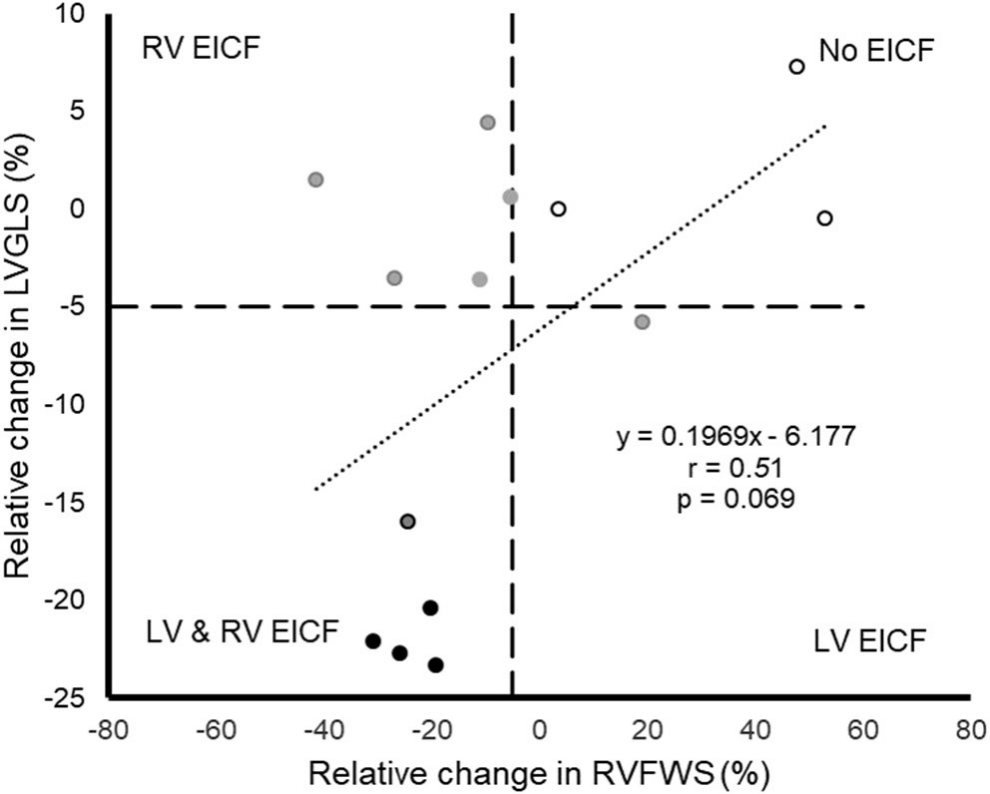
Scatterplot displaying the individual relative change in left ventricular global longitudinal strain (LVGLS) and right ventricular free wall stress (RVFWS) from baseline to post-race in those participants with available data for both measures (*n* = 14). Gray dots represent individuals with exercise-induced cardiac fatigue (EICF) attending to either LVGLS or RVFWS, black dots represent individuals with EICF attending to both LVGLS and RVFWS, and white dots represent individuals without EICF (the criterion for EICF is a decrease greater than 5% in either LVGLS or RVFWS, with the cutoff value marked with dashed horizontal and vertical lines).

**Figure 3:**
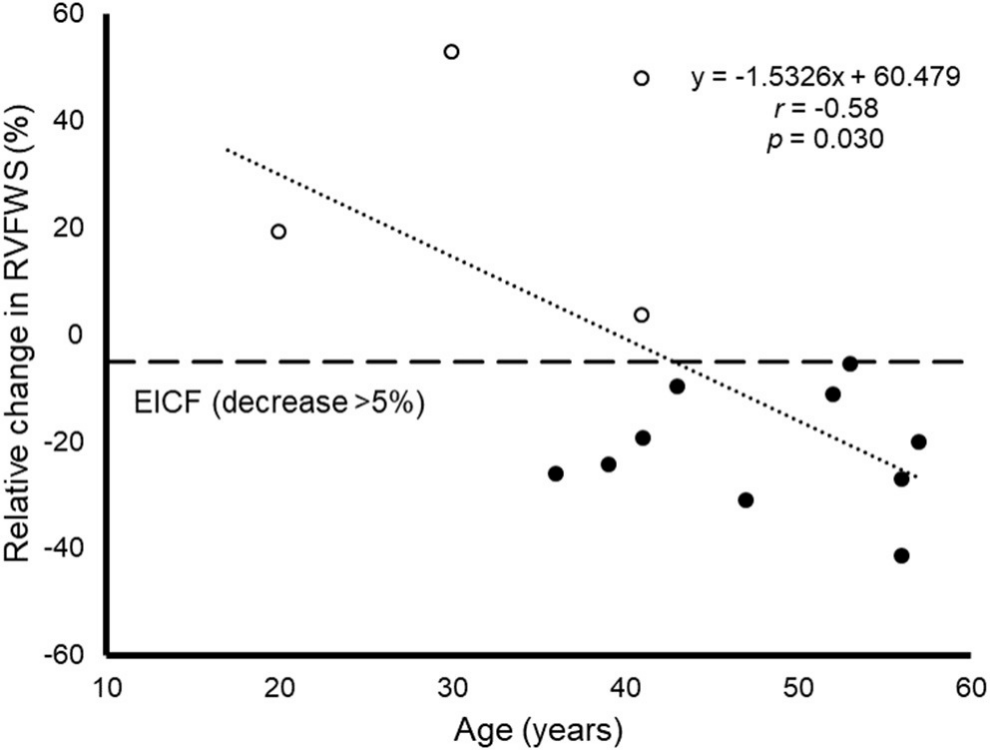
Scatterplot displaying the association between age and the relative decrease in right ventricular free wall strain (RVFWS) from baseline to post-race, which is used as a marker of exercise-induced cardiac fatigue (EICF) of the right ventricle, with EICF defined as a RVFWS decrease of at least 5% and marked with a horizontal line. Black and white dots represent individuals with and without EICF, respectively and the cutoff value of −5% is marked with a dashed horizontal line.

A quasi-significant association was found between the relative change of LVGLS and that of RVFWS (*r* = 0.51, *p* = 0.069) (Figure 2). No significant associations were found between any of the demographic, training or biochemical variables, on the one hand, and risk of EICF (Supplementary Table 1) or relative decrease in LVGLS or RVFWS (Supplementary Table 2), on the other, with the exception of age and RVFWS. Indeed, a significant association was found between age and the magnitude of RVFWS decrease from baseline to post-race (β = 1.53, 1.17 to 2.89, *p* = 0.030, Figure 3). In addition, a non-significant trend (Fisher's exact test *p* = 0.085) was found toward a higher proportion of EICF in the RV among participants aged ≥45 years vs. their younger referents (i.e., 100 and 50% in those aged ≥45 and <45 years, respectively).

## Discussion

The main findings of the present study were three-fold. First, ultraendurance running induced EICF in a considerable proportion of recreational runners, particularly with regard to RV (i.e., present in more than two thirds of cases). There was, however, considerable interindividual variability—especially for the RVFWS response—not only between subjects but also within subjects (i.e., with 43% subjects showing EICF in one ventricle but not in the other). In fact, a significant reduction was found for mean values of LVGLS after the race but not for RVFWS. Finally, an older age was the only potential predictor for an increased risk of EICF, and only so with regard to the RV. Nevertheless, the latter finding must be viewed with caution given the limited number of observations.

There is evidence for a reduction in LV function after strenuous exertion, particularly—but not only—after ultraendurance exercise (>10 h) (10, 17). For instance, Vitiello et al. reported a reduction in LVGLS after 3 h of cycling in healthy young men (9), Lord et al. found a decrease of LV and RV systolic and diastolic function after a 100-mile run in elite runners (10), and Jouffroy et al. (18) reported decrements in LVEF and LVGLS after an 80-km ultraendurance race in recreational runners. Interestingly, in the Jouffroy et al. study the changes in LVEF were only detected at the end of the race whereas LVGLS alterations were already present in the measurements taken after 21 and 53 km, which might support the highest sensitivity of myocardial deformation measures to identify EICF compared to more traditional echocardiographic parameters (6, 7). Other authors, however, have not found changes in LVGLS after endurance or ultraendurance running races (with distances ranging from 25 to 160 km) (19). It has also been suggested that the RV might be more affected than the LV. A classic study by La Gerche et al. found a reduction in integrated systolic strain—particularly of the RV—after an ultraendurance triathlon in athletes of various ages and competition levels (from novices to professionals) whereas the reduction in LVEF did not reach statistical significance (8). The authors suggested that the RV seems less capable than the LV at compensating for the sustained massive CO demanded by prolonged physical efforts. It must be noted, nonetheless, that other authors have found no changes in biventricular function—including both LVEF and strain measures—after ultraendurance exercise in master athletes (14).

The mechanisms underlying EICF are likely multifactorial—and may differ between individuals and also between the RV and LV of the same person—with several influencing factors proposed, including β-adrenergic receptor desensitization, oxidative stress, impaired calcium metabolism, or altered post-exercise loading (11). One mechanism potentially explaining the reduction in LVEF after exercise is a reduction in the cardiac preload, which according to the Frank-Starling mechanism could induce LVEF depression. Nonetheless, strain measures such as those used here are independent of loading conditions and in fact, CO did not significantly decrease after the race in our study. Thus, other mechanisms apart from a reduction in cardiac preload might induce EICF. In this regard, a potential explanation for exertional decreases in LVEF and LVGLS is an intrinsic depression of the inotropism of the heart due to a desensitization of β-adrenoreceptors (as observed after the Hawaii Ironman) (20). Other studies suggest that EICF might be related to myocardial damage, which is supported by the typical elevation of cardiac biomarkers after long and intense exercise (21, 22). However, some authors have proposed that cell death is not responsible for these increases, but rather myocardial stunning—that is, a transient damage to the cell membrane after strenuous exercise with extravasation of cytoplasmic proteins (23, 24). Notwithstanding, we failed to observe a drop in CO (as estimated by AFI) post-effort, which would imply that the level of EICF would not negatively affect tissue perfusion.

Some authors have reported acute RV dilation and dysfunction after intense and prolonged exercise (4, 25). This is believed to be due, at least partly, to the inability of the lung circulation—as opposed to the systemic circuit—to decrease pulmonary vascular resistance (PVR) even under normoxic conditions, which in turn increases pulmonary artery pressure (PAP) and exposes the RV to a greater wall stress compared to the LV (26, 27). In support of this, we found high values of isovolumic relaxation time of the RV in all athletes evaluated after the race, which would correlate with an increase in PVR. However, we did not find a significant decrease in average RVWFS values and in fact there was considerable interindividual variability in the response of this variable. In this context, it has been reported that some athletes can recruit anatomical arteriovenous shunts or to dilate the pulmonary capillaries during exercise, as reflected by the passage of contrast (agitated saline solution) to the left chambers without the presence of an intracardiac shunt, in the immediate post-effort (28, 29). This capacity shown by some individuals is an indicator of pulmonary capillary reserve and is associated with a lower elevation in PAP and a lower stress to the RV wall (29), and would thus explain why RVWFS values do not decrease after strenuous endurance exercise in a number of athletes.

Further research is needed to elucidate if moderate-mild hypoxia like in the present study (i.e., altitude ranging from 1,800 to 3,200 m a.s.l., with the latter decreasing the inspired oxygen pressure by more than 30% compared to sea level) imposes an additional stress in the cardiac tissue—thereby increasing the risk of EICF. Under hypoxic conditions, less oxygen is bound to hemoglobin, which will therefore increase the demand on the cardiovascular system. In addition, hypoxia (*e.g*., 3,000 m a.s.l.) has been shown to induce vasoconstriction of the pulmonary vasculature, leading to further increases in PVR compared to normoxia, and thus potentially increasing PAP and also RV wall stress (28, 29). In this effect, exercising in hypoxic conditions can lead to even greater PAP and RV wall stress, thereby potentially further increasing the risk for EICF in the RV (27–30). However, recent research by Kleinnibbelink et al. does not support that acute exposure to hypoxia (equivalent to 3,000 m a.sl.) affects the magnitude of exercise-induced cardiac fatigue in the LV or RV of untrained individuals compared to normoxia, at least after a bout of endurance exercise of higher intensity than in the present study but of considerably shorter duration (i.e., 45 mins) (11). Although the authors were, like us, unable to directly measure PAP, their findings suggest the presence of an elevated RV wall stress under hypoxic exercise. As such, changing cardiac workload (i.e., from normoxia to hypoxia) does not necessarily change the magnitude of EICF in the RV and may not be the principal mechanism of this condition. One potential explanation for the lack of an impact of hypoxia on EICF shown by Kleinnibbelink and colleagues might be that the loading conditions under hypoxia are not sufficient up to ~3,000 m a.s.l. and/or a longer exposure time is needed to significantly raise RV afterload and thus contribute to the EICF magnitude. On the other hand, although there are also indications that hypoxia itself could induce cardiac dysfunction due to sustained low oxygen availability, this seems to occur mainly during prolonged exposure (i.e., days) at higher altitude (i.e., ≥ 5,000 m. a.s.l.) (31).

Few studies have attempted to elucidate the factors associated with the risk for EICF or with the magnitude of this condition. Some studies suggest that both exercise intensity and duration can increase the magnitude of EICF (17, 18, 32, 33). More controversy exists, however, on how other variables such as training status, dehydration level or age could influence risk of EICF. Although a greater training volume or intensity could theoretically prepare athletes to undergo ultraendurance exercise without suffering EICF, we found no association between different markers of training volume or intensity, and EICF. In this regard, La Gerche et al. found reductions in RV function after an Ironman triathlon in all the athletes they studied, despite the great range in competition level among the study participants (8). On the other hand, and in line with our findings, body weight loss during a 24-h race has shown no association with the magnitude of impairment in cardiac function among experienced male runners (32). Interestingly, however, we observed that an older age was associated with a greater decrease in RVFWS, with all participants above aged 45 years or older presenting EICF in the RV. The changes in cardiovascular function induced by the aging process have been studied mostly in the context of the effects of an advanced age on the LV [e.g., increases in LV wall thickness resulting in a lower compliance and end-diastolic filling, and thus reducing stoke volume, CO, and early diastolic filling while increasing LV diastolic pressures (34, 35), together with reduced β-adrenergic responsiveness (36)]. In fact, there seems to be no evidence for chronic RV damage/dysfunction in elite endurance master athletes with lifelong high training volumes (37). Furthermore, a recent study detected no case of exercise-induced RV—or LV—dysfunction in master endurance athletes (>40 years) after running an ultramarathon (14). More research is thus needed to elucidate the mechanisms explaining the potential association between age and risk of EICF.

Some limitations of the present study should be acknowledged, notably the relatively small number of participants and the lack of subsequent assessments after the race such as to determine to what extent EICF is a transient phenomenon. Another potential caveat stems from the fact that we did not measure some specific markers of cardiac damage, notably circulating cardiac troponin (c-Tn). In this regard, although serum levels of cTn (either cTn-I or c-Tn-T), a marker of injury in acute coronary syndrome, are frequently elevated 24 h post-marathon in amateur runners (38) this transient increase in cTn probably reflects the cytosolic release of the biomarker, which is due to altered cardiomyocyte metabolism (39), not true necrosis, as suggested by a study using delayed enhancement cardiac magnetic resonance imaging (40). Previous research has also shown that marathon-induced elevations in cTn are not associated with cardiac functional changes, inflammation or fibrosis (41). In turn, having conducted all assessments in the context of an ultraendurance race performed at high altitude and having measured a variety of potential predictors including training-related variables (e.g., training volume and intensity) can be considered a major novelty. We also studied a model of intense, demanding ultraendurance as reflected by the very high post-race levels of muscle damage (i.e., CK) and lactate.

In summary, ultramarathon running held at mild-moderate altitude (1,800–3,200 m) seems to induce EICF in a considerable proportion of recreational athletes, with the decrease in RV function being overall of greater magnitude among older ones. In turn, the training status of the participants or other factors such as race time or dehydration level were not associated with EICF. Further evidence is needed to determine the time course and potential sequelae of EICF, as well as to assess other potential predictors or protective factors.

## Data Availability Statement

The datasets presented in this article are not readily available because we cannot share individual data. Requests to access the datasets should be directed to alejandro.lucia@universidadeuropea.es.

## Ethics Statement

The studies involving human participants were reviewed and approved by Universidad Europea Miguel de Cervantes (CEI_2019_001). The patients/participants provided their written informed consent to participate in this study.

## Author Contributions

SW, JP, EG-D, PM, and DW conceived the original idea, designed the study, and collected the data. PLV performed the statistical analysis. PLV and AL drafted the manuscript. AL and AB supervised the study. All authors helped in the interpretation of the data, revised the manuscript critically for important intellectual content, and approved the final version to be submitted.

## Funding

Research by AL is funded by the Spanish Ministry of Economy and Competitiveness and Fondos Feder [AL, Grant PI18/00139]. PLV is funded with a postdoctotal contract from Instituto de Salud Carlos III (Sara Borrell, #CD21/00138). Funders had no role in study design; in the collection, analysis and interpretation of data; in the writing of the report; nor in the decision to submit the article for publication.

## Conflict of Interest

The authors declare that the research was conducted in the absence of any commercial or financial relationships that could be construed as a potential conflict of interest.

## Publisher's Note

All claims expressed in this article are solely those of the authors and do not necessarily represent those of their affiliated organizations, or those of the publisher, the editors and the reviewers. Any product that may be evaluated in this article, or claim that may be made by its manufacturer, is not guaranteed or endorsed by the publisher.
