# Supplementary material for: Exercise-Induced Cardiac Fatigue in Recreational Ultramarathon Runners at Moderate Altitude: Insights From Myocardial Deformation Analysis
Source: Front Cardiovasc Med. 2022 Jan 24;8:744393. doi: 10.3389/fcvm.2021.744393 (PMC8818846; doi:10.3389/fcvm.2021.744393)
Supplement: Supplementary file 1 [file Table_1.DOCX]

**Supplementary Table 1.** Association between different variables and the occurrence of exercise-induced cardiac fatigue (EICF) in the right (RV) and left ventricle (LV) as a dichotomous variable.

| **Variable** | **EICF, RV** | **EICF, LV** |
| --- | --- | --- |
| BMI (kg/cm^2^) | 1.11 (0.71, 1.75)  p=0.647 | 0.81 (0.58, 1.15)  p=0.240 |
| Training experience (years) | 1.23 (0.84, 1.80)  p=0.297 | 1.01 (0.88, 1.16)  p=0.907 |
| Training volume (hours/week) | 0.76 (0.49, 1.15)  p=0.194 | 0.98 (0.75, 1.28)  p=0.876 |
| Training volume (km/month) | 1.01 (0.99, 1.04)  p=0.377 | 1.01 (0.99, 1.03)  p=0.446 |
| Training altimetry  (m a.s.l./session) | 1.00 (1.00, 1.00)  p=0.315 | 1.00 (1.00, 1.00)  p=0.586 |
| Training HR (%HRmax) | 0.98 (0.76, 1.27)  p=0.881 | 0.91 (0.69, 1.19)  p=0.466 |
| Race HR  (%HRmax) | 2.75 (0.07, 115.55)  p=0.596 | 0.93 (0.67, 1.31)  p=0.692 |
| Race finishing time (min) | 1.02 (0.99, 1.04)  p=0.155 | 0.99 (0.97, 1.00)  p=0.128 |
| Body weight loss during the race (%) | 0.93 (0.56, 1.54)  p=0.779 | 1.12 (0.78, 1.59)  p=0.542 |
| CK (U/L) | 1.00 (1.00, 1.00)  p=0.426 | 1.00 (1.00, 1.00)  p=0.417 |
| CK-MB (U/L) | 0.98 (0.94, 1.03)  p=0.476 | 1.02 (0.97, 1.08)  p=0.412 |
| Lactate (mmol/L) | 1.07 (0.39, 2.94)  p=0.891 | 1.45 (0.67, 3.11)  p=0.345 |

Data are odds ratio and 95% confidence interval. Training and race HR could not be obtained in 7 of the 19 participants due to technological issues, whereas blood variables could not be obtained in 2 of 19 participants. Abbreviations: BMI, body mass index; CK, creatine kinase; CK-MB, creatine kinase myocardial band; HR, heart rate; HRmax, age-predicted maximum heart rate; m a.s.l., meters above sea level

**Supplementary Table 2.** Association between different variables and the relative decrease from baseline to post-race in myocardial deformation (‘strain’) of the right (right ventricular free wall strain, RVFWS) and left (left ventricular global longitudinal strain, LVGLS) and right ventricle, both expressed as a continuous variable, from baseline to post-race.

| Variable | **Decrease in RVFWS** | **Decrease in LVGLS** |
| --- | --- | --- |
| Age (years) | **1.53 (0.17, 2.89)**  **p=0.030** | -0.05 (-0.48, 0.38)  p=0.802 |
| Age (≥45 years) | 3.40 (-2.88, 9.69)  p=0.261 | -0.51 (-2.24, 1.22)  p=0.542 |
| BMI (kg/cm^2^) | 0.64 (-1.54, 2.83)  p=0.532 | 0.21 (-0.61, 1.03)  p=0.590 |
| Training experience (years) | -3.34 (-7.51, 0.82)  p=0.106 | -0.41 (-1.91, 1.09)  p=0.575 |
| Training volume (hours/week) | 0.18 (-0.17, 0.53)  p=0.276 | -0.01 (-0.11, 0.10)  p=0.915 |
| Training volume (km/month) | 0.00 (-0.04, 0.03)  p=0.897 | 0.00 (-0.01, 0.01)  p=0.363 |
| Training altimetry  (m a.s.l./ session) | -1.14 (-5.82, 3.57)  p=0.593 | -0.63 (-1.98, 0.72)  p=0.322 |
| Training HR (%HRmax) | 0.49 (-6.21, 7.18)  p=0.871 | 0.07 (-1.71, 1.84)  p=0.936 |
| Race HR  (%HRmax) | 0.12 (-0.15, 0.39)  p=0.354 | 0.00 (-0.08, 0.08)  p=0.987 |
| Race finishing time (min) | -0.95 (-8,47, 6.58)  p=0.789 | 0.61 (-1.29, 2.50)  p=0.509 |
| CPK (U/L) | -0.02 (-0.06, 0.02)  p=0.267 | 0.01 (-0.01, 0.02)  p=0.561 |
| CK-MB (U/L) | -0.17 (-0.80, 0.45)  p=0.559 | 0.08 (-0.18, 0.34)  p=0.510 |
| Lactate (mmol/L) | -2.70 (-14.58, 9.18)  p=0.627 | 2.83 (-1.20, 6.86)  p=0.156 |

Data are β and 95% confidence interval. Significant p-value is in bold. Training and race HR could not be obtained in 7 of the 19 participants due to technological issues, whereas blood variables could not be obtained in 2 of 19 participants. Abbreviations: BMI, body mass index; CK, creatine kinase; CK-MB, creatine kinase myocardial band; HR, heart rate; HRmax, age-predicted maximum heart rate; m a.s.l., meters above sea level
